# Completion of Hepatitis C Virus Replication Cycle in Heterokaryons Excludes Dominant Restrictions in Human Non-liver and Mouse Liver Cell Lines

**DOI:** 10.1371/journal.ppat.1002029

**Published:** 2011-04-28

**Authors:** Anne Frentzen, Kathrin Hüging, Julia Bitzegeio, Martina Friesland, Sibylle Haid, Juliane Gentzsch, Markus Hoffmann, Dirk Lindemann, Gert Zimmer, Florian Zielecki, Friedemann Weber, Eike Steinmann, Thomas Pietschmann

**Affiliations:** 1 Division of Experimental Virology, TWINCORE, Centre for Experimental and Clinical Infection Research; a joint venture between the Medical School Hannover (MHH) and the Helmholtz Centre for Infection Research (HZI), Hannover, Germany; 2 Institute of Virology, University of Veterinary Medicine, Hannover, Germany; 3 Institute of Virology, Carl Gustav Carus Medical Facility, Technical University Dresden, Dresden, Germany; 4 Institute of Virology and Immunoprophylaxis (IVI), Mittelhäusern, Switzerland; 5 Institute of Virology, Philipps University Marburg, Marburg, Germany; The Rockefeller University, United States of America

## Abstract

Hepatitis C virus (HCV) is hepatotropic and only infects humans and chimpanzees. Consequently, an immunocompetent small animal model is lacking. The restricted tropism of HCV likely reflects specific host factor requirements. We investigated if dominant restriction factors expressed in non-liver or non-human cell lines inhibit HCV propagation thus rendering these cells non-permissive. To this end we explored if HCV completes its replication cycle in heterokaryons between human liver cell lines and non-permissive cell lines from human non-liver or mouse liver origin. Despite functional viral pattern recognition pathways and responsiveness to interferon, virus production was observed in all fused cells and was only ablated when cells were treated with exogenous interferon. These results exclude that constitutive or virus-induced expression of dominant restriction factors prevents propagation of HCV in these cell types, which has important implications for HCV tissue and species tropism. In turn, these data strongly advocate transgenic approaches of crucial human HCV cofactors to establish an immunocompetent small animal model.

## Author Summary

The barriers preventing viral transmission across species are only incompletely understood. On one hand, narrow tropism reflects dependence of viruses on host cell factors and their species-specific utilization. On the other hand, host cellular antiviral factors can preclude virus replication. These may be constitutively expressed or induced by interferon triggered upon viral infection. Although viruses have evolved strategies to cope with these “restriction factors” in their natural hosts, these mechanisms often fail in alternative host species. Consequently, restriction factors pose an important barrier to cross-species viral transmission. We investigated if virus-induced or constitutively expressed dominant restriction factors preclude HCV propagation in non-liver tissue and in non-human cells. Using cell fusions between human and mouse cells we show that HCV completes its replication cycle in these heterokaryons. Moreover, we show that the mouse cells analyzed by us are able to sense viral infection and to respond to exogenous interferon. These results rule out that constitutive or virus-induced dominant restriction factors preclude HCV propagation in these cells. These findings have implications for HCV tissue and species tropism and they raise hopes for development of immunocompetent small models for HCV by transgenesis of essential human factors and without manipulation of innate immune mechanisms.

## Introduction

HCV is an enveloped virus that at present chronically infects about 130 million people worldwide [1]. It possesses a positive strand RNA genome of about 9.6 kb composed of non-translated regions (NTRs) at the 5′and 3′ termini required for translation and RNA replication and a single open reading frame encoding a large polyprotein [2]. One hallmark of HCV is its high degree of sequence variability which likely contributes to its ability to establish chronic infections. Different patient isolates are grouped into 6 major genotypes and more than 100 subtypes within the genus *Hepacivirinae* of the family *Flaviviridae* [3]. Persistent infection is associated with a variable degree of liver damage often progressing in severity over the course of decades. Accordingly, a large number of patients are at risk of severe sequelae including life threatening conditions like cirrhosis and hepatocellular carcinoma [4]. The best available treatment, a combination of polyethylene glycol (PEG)-conjugated interferon alpha (IFN-α) and ribavirin, is effective in only a fraction of patients and associated with severe side effects [5]. A prophylactic or therapeutic vaccine is not available.

HCV displays a distinct and narrow tissue and host species tropism and naturally infects only humans and chimpanzees. The latter therefore are the only reliable immunocompetent animal model. However, their use is limited due to ethical reasons, high costs and restricted availability [6]. Transgenic mice containing human liver xenografts have been described and are permissive for HCV infection [7], and summarized in [8]. However, these animals are immunodeficient and hence cannot be used to analyze HCV pathogenesis and immune control or for vaccine development [9].

The mechanisms underlying the restricted tropism of HCV are poorly characterized and likely reflect specific host factor requirements for viral entry, RNA replication, assembly and release. It has been shown that two of the four HCV receptors, namely CD81 and occludin (OCLN) are used in a highly species-specific manner and that the rodent orthologs limit viral entry [10], [11]. Furthermore, mouse scavenger receptor class B type I (SR-BI) and also mouse claudin-1 (CLDN1) were reported to support HCV infection with slightly lower efficiency compared to their human orthologs [12], [13]. Importantly, ectopic expression of all four human entry factors or a combination of mouse SR-BI and CLDN1 with human CD81 and OCLN rendered non-human cell lines permissive for HCV cell entry in tissue culture [10]. Moreover, very recently HCV was adapted in tissue culture to efficiently utilize mouse CD81 as receptor which permitted viral entry into mouse cells in the absence of human entry factors [14]. Together, these results indicate that transgenesis or viral adaptation may be sufficient to overcome the barrier to HCV cell entry also *in vivo*.

HCV RNA replication has been observed in human non-hepatic and murine cell lines. However, the efficiency was very low and required long-term selection procedures using HCV replicon constructs expressing dominant antibiotic-selectable markers [15], [16], [17], [18], [19]. Furthermore, assembly and release of new progeny viruses has so far only been described in cells of human liver origin and was not observed in mouse cells [20]. Presently, it is not clear if low replication efficiency and lack of virus production in these cell types are caused by the absence of crucial viral cofactors, by genetic incompatibility of essential host factors or alternatively by expression of dominant restriction factors interfering with replication and virus production in these cells.

Notably, a number of host-encoded restriction factors which protect mammalian cells from viral infections, particularly by retroviruses, have been recognized [21], [22]. Well characterized examples are APOBEC3G [23], TRIM5-α proteins [24], and tetherin [25] that inhibit human immunodeficiency virus 1 (HIV-1) propagation at various steps of its replication cycle. In case of HCV, EWI-2wint, a CD81-binding protein, impedes HCV entry by competing with the viral glycoproteins for interaction with CD81 in several non-hepatic cells [26]. Moreover, VAP-C which is highly expressed in various non-hepatic tissues but was not detected in human liver from chronically infected HCV patients negatively regulates HCV RNA replication thus possibly contributing to the hepatotropism of HCV [27].

Given these findings, we developed *trans*-complementation systems to investigate if constitutive or virus-induced expression of dominant restriction factors precludes HCV propagation in human non-hepatic tissues or non-human liver cells.

## Results

### Inefficient replication and virus production in human non-hepatic and mouse liver cell lines

**Figure 1:**
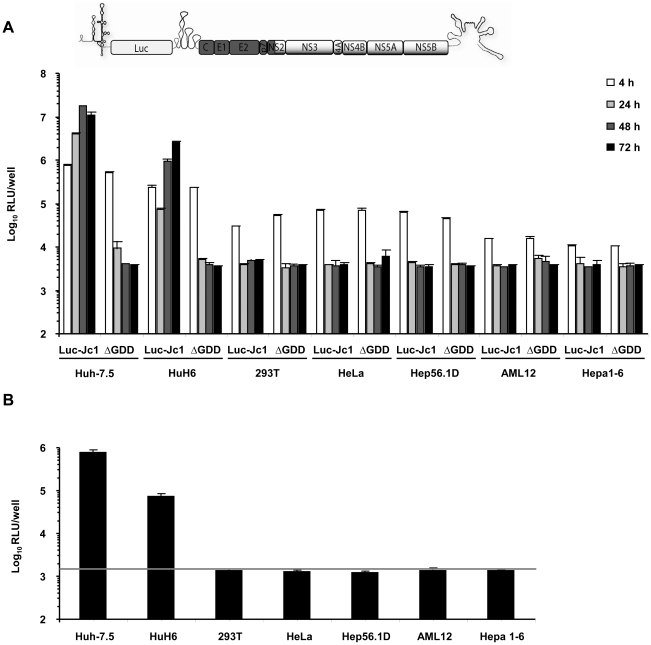
HCV RNA replication and virus production in transfected human and mouse cell lines. (A) Different human and mouse cell lines were transfected with Luc-Jc1 or a replication-deficient construct Luc-Jc1ΔGDD. HCV RNA replication was determined 4, 24, 48, 72 h post transfection using luciferase assays and is expressed as relative light units. (B) In parallel, cell-free culture fluid of transfected cells was harvested 48 h post transfection and infectivity was determined by inoculation of naïve Huh-7.5 cells with cell supernatants and subsequent luciferase assays at 72 h post inoculation. Mean values of quadruplicate measurements of a representative experiment with 3 independent repetitions are shown. The grey horizontal bar represents the background luciferase activity determined in mock infected Huh-7.5 cells.

Given previous reports that HCV replication is inefficient in human non-liver and mouse liver cells and that virus production does not occur, we first quantitatively assessed the extent of HCV RNA replication and virus production in a number of cell lines of human non-liver origin and in mouse liver cell lines. To this end, we transfected 293T (human embryonic kidney), HeLa (human cervix carcinoma), and the mouse liver cell lines Hep56.1D, AML12, and Hepa1-6 with Luc-Jc1 RNA, a highly efficient genotype 2a chimeric genome containing a luciferase reporter gene [28]. Human liver cell lines Huh-7.5 [29] and HuH6 [30] were transfected as reference. Transient viral RNA replication was monitored by quantification of luciferase activity between 4 and 72 h post transfection (Figure 1A). A non-replicative mutant genome with a deletion of the conserved GDD motif within the NS5B RNA dependent RNA polymerase (ΔGDD) was used as control. In parallel we quantified production of infectious virions by inoculation of the highly permissive human hepatoma cell line Huh-7.5 with the culture fluid of the transfected cells and subsequent determination of luciferase activity (Figure 1B). When we transfected Huh-7.5 cells with Luc-Jc1, luciferase activity rose to more than 10^7^ relative light units (RLU) per well at 48 h surpassing reporter activity measured in ΔGDD-transfected cells by more than three orders of magnitude. Similarly, transfection of HuH6 cell yielded high luciferase activity 72 h post transfection, thus confirming highly efficient RNA replication of this HCV genome in these human liver cell lines. In contrast, luciferase activity in transfected human non-liver and non-human cells was indistinguishable between replication-competent Luc-Jc1 and the ΔGDD mutant suggesting that RNA replication did not occur or was too inefficient for detection by this assay. Of note, reporter gene activity 4 h post transfection was lower in these latter cell lines compared to Huh-7.5 and HuH6 cells indicating that either transfection efficiency had been poor or that RNA translation was low in these cells. However, when delivering the viral RNA by lipofection rather than electroporation we reached comparable levels of luciferase activity between all cell lines and more than 1,000-fold above the background at 4 h post transfection. Nevertheless, at subsequent time points post transfection there was again no difference between Luc-Jc1 and ΔGDD (data not shown), thus excluding that merely low transfection or translation efficiency was responsible for absent or inefficient RNA replication. Release of infectious particles was only observed from Huh-7.5 and HuH6 cells transfected with Luc-Jc1, but not from the other cell lines tested (Figure 1B). Collectively, these results are in line with previous observations of a limited HCV RNA replication efficiency in human non-hepatic and mouse liver cell lines.

### Efficient *trans*-complementation of HCV assembly and release in heterokaryons between human liver cell lines

**Figure 2:**
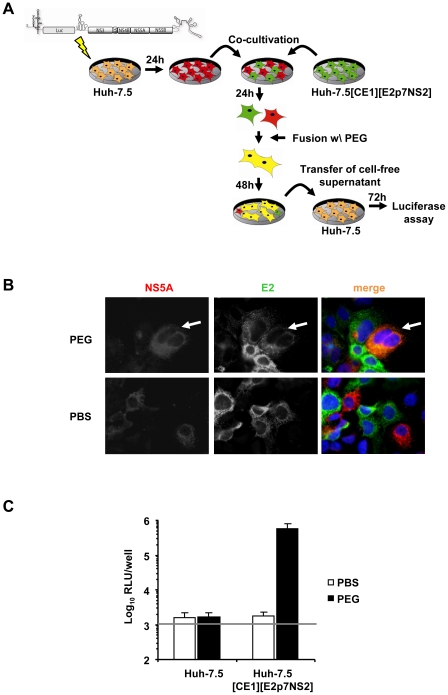
*Trans*-complementation of HCV assembly and release in heterokaryons between human liver cells. (A) Schematic overview of the experimental procedure of PEG-mediated cell fusion. The JFH1 luciferase reporter replicon Luc-NS3-5B was transfected into naïve Huh-7.5 cells. The next day cells were washed with PBS and co-cultured with naïve Huh-7.5 cells or Huh-7.5 packaging cells constitutively expressing core, E1, E2, p7 and NS2 (Huh-7.5[CE1][E2p7NS2]). After 24 h of incubation, fusion was induced by treating co-cultivated cells with 40% PEG for 5 min at 37°C. Treatment with PBS instead of PEG served as control. After 48 h cell-free supernatant was used to inoculate naïve Huh-7.5 cells following determination of infectivity by luciferase assay. (B) For the detection of cell fusion and heterokaryon formation, cells were immunostained using monoclonal antibodies directed against structural protein E2 and non-structural protein NS5A. Simultaneous expression of both proteins within cells is indicative of heterokaryon formation (compare white arrows). (C) Fusion of Huh-7.5 cells transfected with a subgenomic reporter replicon to either Huh-7.5 naïve or Huh-7.5 packaging cells using PEG or PBS as a negative controls. Production of infectious particles was quantified by inoculation of naïve Huh-7.5 cells followed by luciferase readout 72 h post-infection. Mean values of 4 independent experiments and the standard deviations of the means are given. The grey horizontal bar represents the background luciferase activity determined in mock infected Huh-7.5 cells.

To explore if one or more steps of the HCV life cycle are inhibited by expression of host factors that dominantly restrict HCV, we first developed a system to measure dominant restrictions of HCV assembly and release. To this end Huh-7.5 cells were transfected with an assembly-defective subgenomic JFH1 replicon carrying a luciferase reporter gene but lacking the essential viral assembly factors core, E1, E2, p7 and NS2. Subsequently, these cells were co-cultured with Huh-7.5 packaging cells constitutively expressing the aforementioned viral proteins [31]. After induction of cell fusion between these cell types by PEG, *trans*-complementation between replicon and constitutively expressed viral proteins should selectively restore virus production in heterokaryons resulting from fusion of replicon with packaging cells (Figure 2A). Heterokaryon formation was monitored by co-localization of NS5A and E2 proteins, expressed from the replicon and the packaging cell, respectively. Finally, virus production was quantified by collecting the culture fluid of the co-cultured cells 48 h after fusion induction and by inoculation of highly permissive Huh-7.5 cells and subsequent luciferase assays. In case of PBS-treated co-cultured cells we did not observe cells simultaneously expressing E2 and NS5A proteins indicating that this kind of treatment did not induce formation of heterokaryons (Figure 2B). In contrast, when co-cultured cells were treated with PEG we readily detected cells expressing both E2 and NS5A proteins thus indicating that cell fusion between the different cell types had occurred. Importantly, cell fusion between these cells efficiently restored virus production and resulted in transduction of luciferase activity to naïve Huh-7.5 cells inoculated with culture fluids collected from PEG-treated co-cultures of replicon and Huh-7.5[CE1][E2p7NS2] packaging cells (Figure 2C). Notably, PBS treatment of these co-cultures as well as PEG treatment of co-cultures between replicon and naïve Huh-7.5 cells did not permit transduction of luciferase activity. Together these results indicate that the *trans*-complementation assay described above permits rescue of HCV particle production in heterokaryons between human liver cells in a cell fusion-dependent fashion.

### Production of infectious HCV from heterokaryons between human liver cell lines and human non-hepatic or mouse liver cell lines

**Figure 3:**
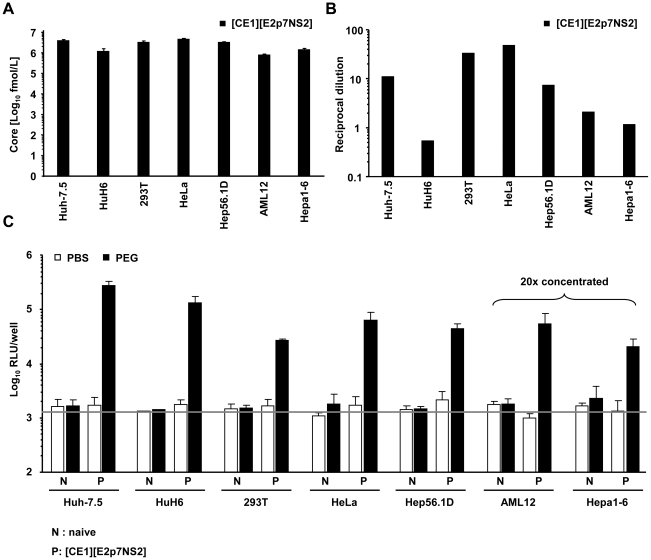
Production of infectious HCV from heterokaryons between human liver cells and human non-hepatic or mouse liver cells. (A) HCV packaging cell lines constitutively expressing core, E1, E2, p7 and NS2 were created using two lentiviral vectors expressing core, E1 and E2, p7, NS2, respectively (Materials and Methods, and see also Table S1). Core protein expression in the individual packaging cell lines as determined by a commercial core-specific ELISA. (B) Lysates of given packaging cell lines were normalized for equal total protein content, serially diluted and incubated with *Galanthus nivalis* lectin coated culture plates to capture glycosylated proteins. Bound viral E2 protein was detected using an E2-specific monoclonal antibody (AP33). In each case, lysates of the parental cell line served as negative control. The OD value was plotted against the reciprocal dilution of the cell lysate. Relative expression of E2 protein between different cell lysates was determined by linear regression analysis as described in Material and Methods. The reciprocal dilution of the cell lysate required to reach an OD value of 0.5 for each lysate is given. Raw data of the ELISA are depicted in Figure S1. (C) Huh-7.5 cells transfected with the JFH1 replicon Luc-NS3-5B RNA were fused to the indicated naïve or packaging cell lines using PEG. Forty eight hours later cell-free media of these cultures were collected and used to inoculate naïve Huh-7.5 cells. Viral infectivity was quantified using luciferase assays. Mean values of 3 independent experiments and the standard deviations of the means are given. The grey horizontal bar represents the background luciferase activity determined in mock infected Huh-7.5 cells. Note that in case of heterokaryons involving AML12 and Hepa1-6 packaging cells, culture fluids were concentrated 20-fold to enhance sensitivity of the assay.

Next, we applied this system to investigate if packaging cell lines from human non-hepatic or mouse liver cells would also rescue HCV particle production when fused with Huh-7.5 replicon cells. To this end we created several novel packaging cell lines by using two lentiviral vectors transducing core, E1 and E2, p7, NS2 proteins, respectively [31] (Table S1). Transgene expression was confirmed by western blot (data not shown) and by using a commercial core specific ELISA as well as an E2-specific ELISA for detection of representative proteins derived from the individual vectors (Figure 3A and 3B and Figure S1). Core protein expression was readily detected and was very similar between all individual packaging cell lines (Figure 3A). Similarly, E2 protein expression was well detectable (Figure 3B) with high E2 protein levels in HeLa and 293T, intermediate amounts in Huh-7.5 and Hep56.1D and comparatively low quantities in AML12, Hepa1-6 and HuH6 packaging cells. Importantly, after co-culture with Huh-7.5 cells transfected with the luciferase replicon and induction of heterokaryons by PEG all packaging cell lines tested efficiently rescued production of infectious HCV particles as is evident from transduction of high levels of luciferase activity to the inoculated naïve Huh-7.5 cells (Figure 3C). Since neither PBS treatment of these co-cultures, nor cell fusion between Huh-7.5 replicon cells and the naïve non-liver or non-human cell lines permitted transduction of luciferase, we concluded that virus production required cell fusion and expression of viral proteins core, E1, E2, p7 and NS2 in the packaging cell lines. Together, these data indicate that infectious HCV particles are produced upon fusion between human liver cells expressing a subgenomic HCV replicon with various human non-liver cells or mouse liver cells providing the remaining viral proteins in *trans*.

**Figure 4:**
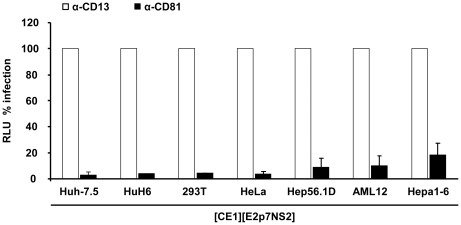
Neutralization of virus particles released from heterokaryons by CD81- specific antibodies. Huh-7.5 cells were inoculated with supernatants derived from heterokaryons between Luc-NS3-5B transfected Huh-7.5 cells and given packaging cell lines in the presence of CD81-specific monoclonal antibodies or CD13 control antibodies. The efficiency of infection was determined 72 h later by luciferase reporter assay and is expressed relative to infection in the presence of CD13-specific antibodies. Mean values of at least two independent experiments and standard deviations of the means are displayed.

To firmly establish that infectious particles produced from these heterokaryons enter Huh-7.5 target cells in the same way as authentic HCV particles and that luciferase expression in the inoculated cells resulted from genuine infection rather than non-specific transfer of reporter gene activity, we investigated if the released particles infect target cells in a CD81-dependent manner. Huh-7.5 cells were inoculated with cell culture fluids harvested from heterokaryon-cultures in the presence of CD81-specific antibodies or control antibodies against CD13, an irrelevant protein expressed on the surface of Huh-7.5 target cells. As depicted in Figure 4, anti-CD81 antibodies reduced reporter activity in inoculated cells by more than 80% to almost background levels (Figure 4).

Taken together, these results show that formation of heterokaryons between Huh-7.5 replicon cells and human hepatic, human non-hepatic and mouse hepatoma packaging cell lines rescued virus production and that produced infectious particles utilize CD81 during infection. Therefore, these data indicate that HCV replication, assembly and release are not repressed in these non-permissive cell lines in a dominant negative fashion.

**Figure 5:**
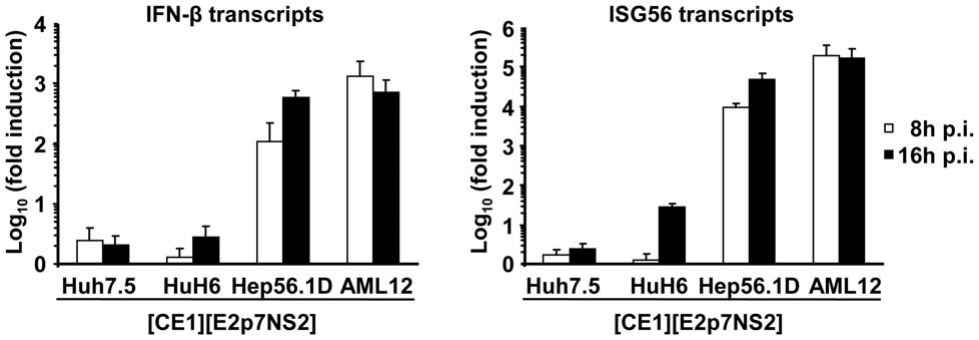
Responsiveness of selected packaging cell lines to viral pathogen associated molecular patterns. Given cell lines were inoculated with a recombinant La Crosse virus lacking the non-structural protein NSs (rLACVdelNSs;[32]). At 8 or 16 h post inoculation (p.i.) cells were collected and expression levels of IFN-β (left) and ISG56 (right) were determined by quantitative RT-PCR. The relative induction compared to cells that were not inoculated with the virus is given. Mean values of three independent experiments including the standard deviations are given.

Notably, the efficiency of virus production between heterokaryons was substantially different (Figure 3C). This could be due to various reasons, including dissimilar expression levels of transgenes (HCV core to NS2 proteins), fusion efficiency, and abundance of replication co-factors or restriction factors. Regarding the latter, kinetic and extent of response to pathogen associated molecular patterns (PAMPs) and subsequent induction of antiviral mechanisms may substantially influence virus production. Therefore, to investigate why heterokaryons between human Huh-7.5 replicon cells and mouse AML12 packaging cells produced more than 20-fold lower infectious virus particles than heterokaryons between Huh-7.5 replicon cells and human HuH6 packaging cells or mouse Hep56.1D packaging cells (Figure 3C), we compared fusion efficiency and responsiveness to viral PAMPs between these packaging cell lines. The relative number of fused cells displaying both replicon-expressed NS5A and at the same time E2 from the lentiviral transduction was similar between these packaging cell lines (Figure S2). Therefore, we can exclude that dissimilar fusion was responsible for these marked differences in virus production. Responsiveness of packaging cell lines to viral PAMPs was measured by inoculation with a recombinant La Crosse virus lacking the non-structural protein NSs (rLACVdelNSs;[32]). Subsequently, mRNA levels of ISG56 and IFN-β were determined by quantitative RT-PCR as a measure of antiviral signaling. As expected, Huh-7.5 packaging cells which carry a defective RIG-I protein [33], that is essential for detection of viral PAMPs, did not upregulate ISG56 or IFN-β mRNA levels (Figure 5). Notably, also HuH6 packaging cells responded poorly and only marginally up-regulated ISG56 and IFN-βmRNA levels upon La Crosse virus challenge. In contrast both mouse packaging cell lines strongly and rapidly increased transcription of ISG56 and IFN-β thus indicating that they efficiently detect viral PAMPs. Given that Hep56.1D packaging cells fused with Huh-7.5 replicon cells readily produce infectious particles despite of vigorous responsiveness to viral PAMPS, these findings exclude that rapid and efficient recognition of viral PAMPs in mouse packaging cell lines precludes production of infectious virus particles in the heterokaryons.

### Both human and mouse IFN-α inhibit virus production in human-mouse heterokaryons

**Figure 6:**
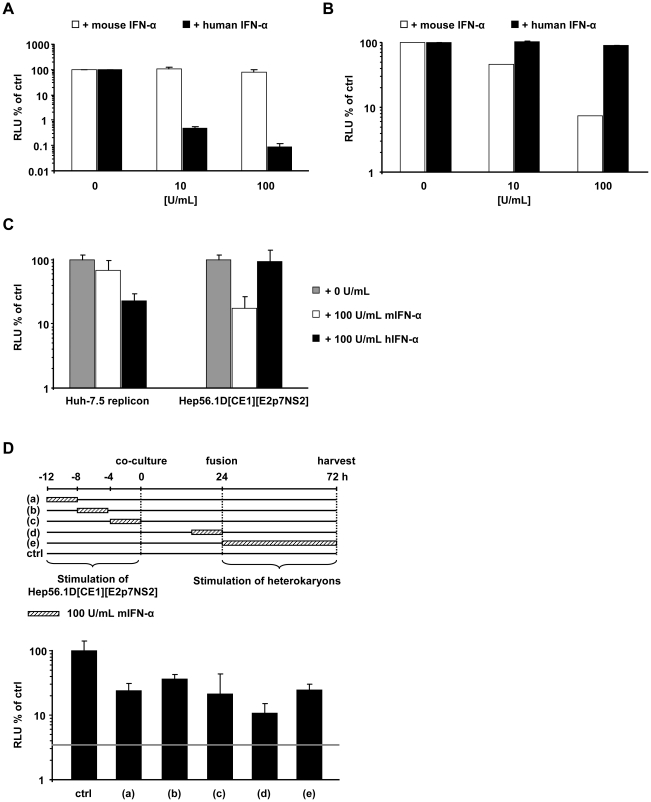
Effect of human and mouse IFN-α treatment on HCV and VSV in human and mouse cells as well as in human-mouse heterokaryons. (A) Four hours prior to infection with Luc-Jc1, Huh-7.5 cells were treated with the indicated dose of human or mouse IFN-α. HCV infectivity was assessed by measuring luciferase activity 72 h post-infection. (B) Hep56.1D cells were pre-incubated for 4 h with different doses of human or mouse IFN-α following inoculation with Luc-VSV for 4 h. After 72 h infectivity was determined by luciferase assay. (C) Either Huh-7.5 replicon cells or Hep56.1D[CE1][E2p7NS2] packaging cells were incubated with human or mouse IFN-α for 4h before co-culture. Production of *trans*-complemented particles after PEG-mediated induction of cell fusion was quantified by inoculation of naïve Huh-7.5 cells with cell supernatants of these cultures and determination of luciferase activity 72 h later. Mean values of two independent experiments and standard deviations of the means are displayed. (D) Mouse IFN-α was applied to Hep56.1D packaging cells for 4 h either at indicated time points before co-culture, or to cells co-cultured with Huh-7.5 replicon cells as indicated by cross hatched boxes of the diagram displayed above. Alternatively, mIFN-α was present for 48 h directly after fusion induction and until harvesting of cell culture fluid. In each case infectivity produced was determined by inoculation of naïve Huh-7.5 cells and is expressed relative to the control which was not treated with mouse IFN-α. Mean values of quadruplicate measurements including the standard deviations are shown. The grey horizontal bar indicates the background of the assay based on the luciferase activity determined in Huh-7.5 cells that had not been infected.

HCV propagation in tissue culture is strongly inhibited by interferon, albeit by as far incompletely defined anti-viral mechanisms. To exclude that Hep56.1D packaging cells sustain virus production due to the inability to respond to IFN, we used mouse interferon alpha (IFN-α) to establish that these cells are not refractory to IFN and that our cell fusion assay efficiently detects dominant restriction factors – in this case IFN induced – that prevent efficient HCV virus production. First we confirmed that mouse IFN-α induces an antiviral state only in mouse cells and not in human cells incubated with recombinant mouse IFN-α. Since Hep56.1D cells cannot be infected with HCV, we chose an interferon-sensitive vesicular stomatitis reporter virus (Luc-VSV) which efficiently infects mouse cells to assess responsiveness of these cells to mouse and human IFN-α. To evaluate sensitivity of Huh-7.5 cells to IFNs of human or mouse origin we employed the HCV reporter virus Luc-Jc1. Importantly, infection of Huh-7.5 cells by Luc-Jc1 was only inhibited by human IFN but not affected by mouse IFN (Figure 6A). In turn infection of Hep56.1D cells by Luc-VSV was only inhibited by mouse IFN but not by human IFN (Figure 6B). Together these results confirm that both cell lines are responsive to IFNs and that induction of the antiviral state by IFN is species-specific in both cell lines. Next we explored if both mouse and human IFN induce restriction factors in Huh-7.5 replicon or Hep56.1D packaging cells which prevents virus production in fused heterokaryons. To this end, Huh-7.5 replicon or Hep56.1D packaging cells were pre-incubated with either human or mouse IFN-α, subsequently these cells were co-cultured and fused by PEG, before production of virus particles was measured by transduction of luciferase activity to naive Huh-7.5 cells (Figure 6C). In this context production of infectious HCV particles was strongly repressed by both mouse and human IFN-α suggesting that both mouse and human IFN-α induced antiviral effector mechanisms that efficiently control HCV particle production.

Finally, to explore the importance of the time point of IFN-treatment for virus production in the heterokaryon fusion assay between Hep56.1D packaging cells and Huh-7.5 replicon cells, we applied mouse IFN-α for 4 h at different time points before co-culture, during co-culture or after induction of cell fusion by PEG (Figure 6D). Interestingly, mouse IFN-α repressed virus production with comparable efficiency irrespective of the time point of addition arguing that the antiviral response induced via mouse IFN-α is relatively sustained thus suppressing virus production at least up to 80 h post treatment.

### Completion of entire virus replication cycle in human-mouse heterokaryons

**Figure 7:**
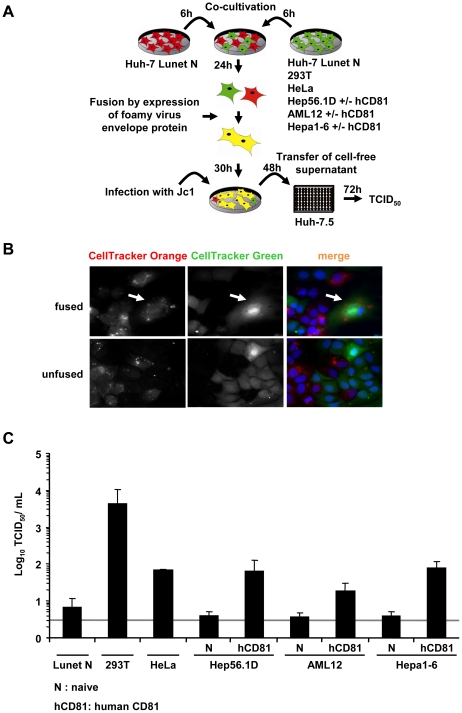
Completion of HCV replication cycle after infection of heterokaryons. (A) Schematic overview of the experimental procedure. Huh-7 Lunet N cells lacking CD81 (refractory to HCV cell entry; [34]) were co-cultured with the indicated cell lines lacking or expressing human CD81 (see also Table S2). Fusion between these cells was initiated by transfection of a fusogenic viral envelope protein derived from the prototype foamy virus the next day. Thirty hours later, cells were challenged with infectious HCV particles. Cell entry, RNA replication and virus production sustained by these cultures was quantified by determining infectivity titers in the cell-free culture fluid 48 h post inoculation using naïve Huh-7.5 cells as target cells for a limiting dilution assay. (B) For the detection of heterokaryons formed due to expression of the prototype foamy virus envelope protein, cells were stained with CellTracker Green or CellTracker Orange 6 h prior to co-cultivation (and transfection). Thirty hours later, cells were fixed with 3% PFA and stained with DAPI. The white arrow indicates heterokaryons produced upon co-culture of Lunet N cells labeled with CellTracker Orange and Lunet N cells stained with CellTracker Green. (C) Huh-7 Lunet N cells were fused to the different naïve cell lines or cell lines transduced to express human CD81. These cultures were inoculated with cell culture derived HCV particles (MOI of 2.3). Culture fluid was collected 48 h later and de novo particle release from heterokaryons was determined by limiting dilution (TCID_50_) using naïve Huh-7.5 target cells. Mean values of 3 independent experiments are given. The grey bar represents the detection limit of the limiting dilution assay.

It is well established that a high burden of viruses can saturate and ultimately overcome endogenous host cell restrictions. To exclude that a high viral load in transfected Huh-7.5 cells may have precluded detection of restrictions present in human non-liver or mouse liver cells simply by saturation of such factors, we wished to confirm our findings using a second independent approach based on HCV infection rather than transfection. To this end we used a Huh-7-derived cell line designated Lunet N which lacks endogenous CD81 expression [34]. As a consequence, Lunet N cells cannot be infected by HCV unless human CD81 is reintroduced [14]. When these cells are fused with cells expressing human CD81 but not the complete set of viral entry factors, only fused cells display all necessary entry factors thus rendering only the heterokaryons susceptible. Following this rationale, we co-cultured Lunet N cells either with 293T or HeLa cells which both express human CD81 but which are non-permissive to HCV cell entry due to lack of at least one essential HCV entry factor [10], [35] (Table S2). In parallel we fused Lunet N cells with naïve Hep56.1D, AML12 or Hepa1-6 cells or derivates of these cell lines expressing human CD81. Prior to co-cultivation, cell lines were stained by two different fluorescent CellTrackers to allow discrimination of cell types and detection of successful cell fusion [36]. Formation of heterokaryons between cells was subsequently induced by a transient transfection of a plasmid encoding a fusogenic viral glycoprotein of the prototype foamy virus [37] (Figure 7A). This method of cell fusion induction was chosen to increase sensitivity of the assay due to increased fusion rates. Fluorescence microscopy revealed that the expression of the highly fusogenic glycoprotein led to the accumulation of syncytia stained with both CellTrackers (Figure 7B). Thirty hours after transfection of the fusogenic envelope protein into the co-cultured cells, the cell population was inoculated with the HCV Jc1 chimera (MOI 2.3) which grows to high virus titers in tissue culture [38]. Completion of the viral replication cycle including productive entry, RNA replication as well as assembly and release of infectious progeny virus particles was measured by titration of cell free infectivity released from these cells 48 h post inoculation. To rule out that residual infectivity of the inoculum or low numbers of directly infected susceptible Lunet N cells are solely responsible for the detection of infectious virus at this time point, we included Lunet N cells fused with Lunet N cells as control. Importantly, in this control we observed only very low levels of infectious HCV ranging between 5 and 10 TCID_50_/mL which is close to the detection limit of the limiting dilution assay. In contrast, when Lunet N cells were fused with 293T cells and challenged with the same viral inoculum, viral titers as high as 4×10^3^ were detected providing strong evidence that in this case relatively high numbers of progeny infectious particles had been produced (Figure 7C). Similarly, when using HeLa cells for heterokaryon formation with Lunet N cells, also substantial numbers of infectious particles were produced, reaching levels approximately 10-fold higher than the background infectivity observed with Lunet N cells. Importantly, HCV inoculation of Lunet N cells fused with mouse liver cells expressing human CD81 also resulted in production of well detectable quantities of infectious HCV about 5- to 20-fold higher than after fusion of naïve mouse liver cells with Lunet N cells (Figure 7C). In conclusion these results indicate that fusion of Lunet N cells with cells expressing human CD81 complements the lacking surface receptor, thus allows HCV cell entry and de novo production of infectious particles from the resulting heterokaryons. Importantly, completion of the HCV replication cycle was observed when human liver cells (Lunet N cells) were fused with human non-liver cells (293T, HeLa) and three different mouse liver cell lines. It is worth mentioning here that all three mouse liver cell lines express functional viral pattern recognition pathways as is evident from efficient induction of IFN-β and ISG56 gene transcription as well as IFN secretion upon stimulation with viral RNA, poly(I:C) or upon infection with a Bunyavirus mutant (Figure S3). Together these findings provide strong evidence that these cell types do not express dominant restriction factors precluding HCV entry, RNA replication, virus assembly and release and that the endogenous pattern recognition pathways are insufficient to fully control HCV propagation.

## Discussion

HCV is a highly tissue- and species-specific virus which efficiently replicates only in human and chimpanzee liver cells. Three principal mechanisms may prevent HCV from infecting other species. These are lack of essential host cell factors, their genetic incompatibility, or alternatively the expression of dominant restriction factors. Previous reports have highlighted that indeed inefficient usage of HCV entry factors from mouse origin precludes infection of mouse cells [10], [11], [12], [13]. Moreover, ectopic expression of a liver-specific miRNA (miR-122) which is important for efficient HCV RNA replication [39] has been shown to facilitate HCV replication in human non-hepatic cell lines or mouse fibroblasts [40], [41]. Finally, it was noted that HCV cell entry is inhibited by expression of a CD81-binding protein in non-liver cells [26]. However, comprehensive studies investigating dominant cellular restriction factors which may limit HCV replication have been lacking.

In case of retroviruses, most notably HIV-1, it is well established that host factors can potently repress completion of the retroviral replication cycle: For instance pro-virus formation of HIV-1 is impeded by APOBEC3G [23], [42], TRIM5-α inhibits HIV-1 by acting before reverse transcription [24], and tetherin prevents release of virions from infected cells [25], [43]. Notably, HIV-1 encodes viral factors like Vif and Vpu that efficiently suppress these innate host cell defense mechanisms by inducing degradation of APOBEC3G and tetherin, respectively [25], [44], [45]. Importantly, viral interference with these factors is species-specific, since for instance Vpu fails to protect HIV-1 from non-human tetherin [45], [46] and as HIV-1 Vif does not interfere with murine APOBEC3G [47], [48]. Consequently, the interaction of HIV-1 with restriction factors plays a key role in determining species tropism (summarized in [49]). Notably, also the hepatitis B virus is targeted by APOBEC3G [50] and tetherin prevents release of diverse enveloped viruses from divergent virus families including retroviruses, arenaviruses, filoviruses and herpesviruses [51], [52], [53], [54].

Given these circumstances we investigated if restriction factors limit HCV propagation in non-permissive cell lines by using somatic cell fusion between permissive and non-permissive cells. Employing this approach we provide strong evidence that HCV efficiently completes its replication cycle in heterokaryons between human liver cells and human non-hepatic or mouse liver cell lines. In turn, these results suggest that HCV propagation in these cell types, and possibly also in primary human non-liver tissue or in mouse liver, is not ablated by constitutive or virus-induced expression of restriction factors. This conclusion is based on two independent experimental systems employing in total five cell lines. We ensured that virus production can only occur in true heterokaryons between human liver cells and non-permissive cell lines by choosing two different *trans*-complementation systems. On one hand, virus production of a subgenomic HCV replicon transfected into highly permissive Huh-7.5 cells was complemented by fusion of these cells with packaging cells constitutively expressing the lacking viral assembly factors. Alternatively, we took advantage of a Huh-7-derived cell clone lacking endogenous CD81 expression and fusion with cell lines expressing human CD81. When challenged with HCV, only heterokaryons between these cell types express all necessary factors rendering only these permissive for viral entry.

Notably, in both systems productive complementation of the viral replication cycle likely depends on a number of factors including transgene expression, efficiency of cell fusion as well as abundance of essential (in part unknown) viral co-factors and viral restriction factors. In part these factors cannot be quantified; therefore it is not possible to establish at this point why virus production efficiency differs between heterokaryons. Notably, we observed much lower virus production in heterokaryons between Huh-7.5 replicon cells and AML12 packaging cells compared to heterokaryons between Huh-7.5 replicon cells and HuH6, or Hep56.1D packaging cells (Figure 3C). Since the fusion efficiency was similar between these cells, we can exclude that this accounts for these dissimilarities in virus production. Moreover, we can rule out that efficient detection of viral PAMPs precludes virus production in these heterokaryons as for instance Hep56.1D cells readily respond to viral triggers of antiviral signaling (Figure 5) but nevertheless produce comparatively high levels of infectious particles in the heterokaryon assay (Figure 3C). It is worth mentioning though that virus production in heterokaryons involving either Huh-7.5 packaging cells or HuH6 packaging cells was consistently 3 to 4 fold more efficient than in those with Hep56.1D packaging cells. Since Hep56.1D packaging cells express at least as much viral transgenes and fuse similarly well as the human liver Huh-7.5- and HuH6-derived packaging cells this difference could be due to either lower abundance of important viral cofactors in the mouse liver cell line or due to partial control of virus production by cellular antiviral defenses in Hep56.1D packaging cells. In any case, our data firmly exclude that dominant restriction factors fully ablate HCV propagation in these cell types. In turn, it is reasonable to assume that lack of essential human co-factors is a primary limitation for efficient HCV propagation in mouse cells. Such species-specific host factors could for instance be identified by introduction of human cDNA libraries into mouse cells and subsequent screening for those cDNAs which confer increased HCV replication capacity to mouse cells.

Our results also provide first evidence that virus-induced antiviral programs in mouse cells may be insufficient to fully control HCV infection and replication: It is unlikely that high viral load, as present after transfection, simply overrides endogenous antiviral pathways since we observed completion of the HCV replication cycle not only upon fusion of HCV transfected cells carrying a high dose of replication-competent viral RNA, but also after infection with HCV. Second, we can exclude that the mouse cells are unable to sense viral infection due to lesions in the pathways triggering interferons since ligands of MDA5 [poly(I:C)] and RIG-I (5′triphosphorylated VSV RNA), as well as Bunyavirus infection efficiently elicited ISG56 and IFN-β gene expression as well as IFN secretion. Finally, we can also rule out that the cells tested by us are non-responsive to IFN, since stimulation of antiviral effectors in Hep56.1D mouse cells via mouse IFN-α strongly inhibited Luc-VSV infection or HCV particle production in mouse-human heterokaryons. Importantly, treatment of either Huh-7.5 replicon cells with human IFN-α or likewise Hep56.1D packaging cell lines with mouse IFN-α at different time points prior to co-culture efficiently reduced virus production in the heterokaryons (Figure 6). These results suggest that not only human factors induced by human IFN but also mouse factors stimulated by mouse IFN are able to control HCV particle production. However, in the context of mouse-human heterokaryons these IFN-dependent antiviral effectors are either not sufficiently activated by HCV or not capable to ablate production of infectious particles upon HCV infection for instance due to incompatibility of mouse antiviral effectors for control of HCV infection/replication. Notably, HCV naturally induces cellular innate immunity through RNA composition-dependent activation of RIG-I [55], [56] and subsequent signaling via IRF-3. However, at least in human liver cells HCV interferes with RIG-I dependent signaling to IRF-3 by NS3-4A-dependent cleavage of the crucial adaptor protein Cardif [55]. Likewise, TLR-3 dependent signaling to IRF-3 is ablated by cleavage of Trif through the viral NS3-4A protease [57]. Since infection of mouse-human heterokaryons with HCV particles apparently did not induce innate defenses to an extent sufficient to control HCV propagation, we speculate that HCV's ability to interfere with the induction of innate immunity may be sufficient to prevent adequate recognition and clearance of virus also by mouse innate immune factors. In this regard it is worth mentioning that Ahlén et al. recently reported that the HCV NS3-4A protease is capable of cleaving mouse RIG-I which may be an essential prerequisite for (partial) viral evasion of innate immune control in mouse liver cells [58]. On the other hand, Lin et al. observed that poor replication of HCV in murine embryonic fibroblasts was facilitated by deletion of interferon regulatory factor 3 (IRF-3) suggesting that IRF-3-dependent mechanisms may partially restrict HCV replication in mouse cells [41]. Clearly more work is needed to fully define the interplay between HCV and mouse innate immunity and to appreciate by which extent it contributes to low replication of HCV in these cells.

Despite successful development of cell culture systems to study the complete HCV replication cycle [59], [60], [61], analysis of mechanisms of virus pathogenesis and immune control as well as vaccine development are severely hampered by the lack of immunocompetent small animal models. The results presented in this study provide strong evidence that dominant restriction factors are unlikely to preclude propagation of HCV in mouse liver cells. This raises the hope that permissive small animal models could be developed without manipulation of innate, intrinsic or adaptive immunity factors. Adaptation of HCV to mouse cells or transgenic expressions of human viral co-factors are promising approaches to ultimately establish urgently needed small animal models for HCV.

## Materials and Methods

### Plasmids

The plasmids pFK-Jc1, pFK-Luc-Jc1, pFKi389Luc-EI/NS3-3′_JFH1_dg have been described earlier [28], [31], [38], The construct Luc-Jc1ΔGDD has an in-frame deletion of 10 amino acids (MLVCGDDLVV) encompassing the GDD motif of NS5B [62]. Lentiviral plasmids pWPI-CE1-BSD, pWPI-E2p7NS2-BSD have been described previously [31]. pWPI-CE1-GUN was created by restriction digest of pWPI-CE1-BSD with BamHI and SpeI and transfer of the insert in the digested vector pWPI-GUN [14]. The plasmids pczHFVenvEM066 and pWPIhCD81BLR have been described previously [14], [63]. Exact cloning strategies are available on request. IFN reporter plasmid Mx1-Luc was described elsewhere.

A recombinant, glycoprotein G gene-deleted VSV replicon expressing firefly luciferase, VSV*ΔG(Luc) has been generated by reverse genetics according to published protocols [64]. A detailed characterization of this vector will be published elsewhere.

### Cell culture

Huh-7.5, HuH6, 293T, HeLa, Hep56.1D, and Hepa1-6 cell lines were maintained in Dulbecco's modified Eagle medium (DMEM; Invitrogen, Karlsruhe, Germany) supplemented with 2 mM L-glutamine, non-essential amino-acids, 100 U/mL penicillin, 100 µg/mL streptomycin and 10% fetal calf serum (DMEM complete). AML12 cells were maintained in DMEM/Ham's F-12 medium supplemented with 10% FCS, 40 ng/mL dexamethasone, 5 µg/mL insulin, 5 µg/mL transferrin, 5 ng/mL selenium, 100 U/mL penicillin and 100 µg/mL streptomycin as described before [65].

Cell lines expressing viral proteins C, E1, and E2, p7, and NS2 from two independent genetic cassettes were created by lentiviral gene transfer as described previously [31]. Lentiviral particles were produced using the plasmids pCMV-ΔR8.74 [66], pcz-VSV-G [67], and derivatives of pWPI, (encoding the genes of interest and in addition either the blasticidin S deaminase of *Aspergillus terreus* or a GFP-ubiquitin-neomycin fusion protein (GUN), conferring resistance against blasticidin or G418, respectively [14]. Parental cell lines were transduced and selected in the presence of either blasticidin (Invivo Gen, San Diego, USA) alone (2.5 µg/mL or 5 µg/mL), or together with geneticin (G418; 750 µg/mL, Life Technologies), as indicated in Table S1. Expression of viral proteins was confirmed by Western blot analysis using α-E2 (AP33), α-core (C7-50) and α-mouse-peroxidase antibodies as reported elsewhere [31]. Murine cell lines expressing human CD81 were created by lentiviral gene transfer as described above using a pWPI derivative [14] and selected using blasticidin. Expression of CD81 was confirmed by FACS analysis using α-CD81 (1.3.3.22, Ancell) and goat α-mouse antibody conjugated to APC (eBioscience, data not shown).

### HCV replication and infection assays

HCV particles and firefly luciferase HCV reporter viruses were generated as reported previously [28]. In brief, plasmid DNA was linearized and transcribed into RNA, which was then electroporated into Huh-7.5 cells. Virus-containing culture fluids of transfected cells were harvested 48 h and 72 h after transfection. Luciferase reporter virus infection assays were carried out and analyzed as described [28]. Wildtype HCV particles were titrated by using a limiting dilution assay [31]. The 50% tissue culture infectious dose (TCID_50_) was calculated based on the methods described by Spearman and Kärber [68], [69].

### Polyethylene glycol-mediated fusion and infectivity assay

Formation of heterokaryons between permissive and non-permissive cell lines was induced by PEG. In detail, 10 µg in-vitro RNA transcript of the luciferase reporter HCV replicon Luc-NS3-5B was transfected into naïve Huh-7.5 cells (6×10^6^ cells) by electroporation; the cells were resuspended in 10 mL DMEM and seeded onto a 10 cm dish. The next day cells were washed with PBS and 5×10^5^ cells were co-cultured with 5×10^5^ Huh-7.5 naïve or packaging cells (cell numbers adjusted to growth efficiency) on a 6-well culture plate. After 24 h of incubation, at a cell density of ∼80%, fusion was induced by treating co-cultivated cells with 40% PEG-1500 in PBS (Roche, Mannheim, Germany) or PBS as a control for 5 min at 37°C, followed by extensive washing, addition of 2 mL DMEM/well and 48 h of incubation. Production of infectious *trans*-complemented particles was determined as described [31]. In case of heterokaryons formed with AML12 and Hepa1-6 naïve and the respective packaging cells, assay sensitivity was enhanced by concentration of the supernatants from 6-well plates by 20-fold using Amicon ultrafiltration devices (Millipore) according to the manufacturer's protocol.

### Induction of antiviral responses by Interferon treatment

To test if IFN-α treatment induces cellular restrictions, Huh-7.5 replicon and Hep56.1D packaging cells were pre-incubated with either human IFN-α-2b (0–100 U/mL, IntronA, Essex Pharma, München, Germany) or mouse IFN-α-1 (0–100 U/mL, PBL InterferonSource, Piscataway, USA) for 4 h prior to co-cultivation. For the time kinetic Hep56.1D packaging cells were pre-incubated with mouse IFN-α-1 (100 U/mL) at indicated time points prior to co-cultivation, 4 h prior to PEG-mediated cell fusion, or 4 h post fusion.

### Fluorescence microscopy

Staining of NS5A was performed by using the 9E10 hybridoma supernatant [59] at a dilution of 1∶2000. Immunostaining of E2 was performed using an E2-specific human monoclonal antibody CBH-23 (kindly provided by Steven Foung, unpublished) at a dilution of 1∶250 in PBS supplemented with 5% normal goat serum. After three times washing with PBS, bound primary antibodies were visualized using goat anti-human or goat anti-mouse IgG–specific secondary antibodies conjugated to Alexa Fluor 488 or Alexa Fluor 546 (Invitrogen, Karlsruhe, Germany) at a dilution of 1∶1,000. DNA was stained with DAPI (4′,6′-diamidino-2-phenylindole dihydrochloride; Invitrogen) for 1 min. Finally, cells were washed three times with PBS, once with water and mounted on glass slides with Fluoromount G (Southern Biotechnology Associates, Birmingham, USA).

Staining of cells using CellTracker (CellTracker Green CMFDA, Cell Tracker Orange CMTMR, Invitrogen) was performed as recommended by the manufacturer. Briefly, cells were incubated with 5 µM or 10 µM of CellTracker Green or Orange, respectively in serum-free media. Medium was changed after 45 minutes and cells were incubated in standard DMEM complete medium for 6 hours.

### Determination of core and E2 expression

Viral transgene expression of the two lentiviral vectors encoding HCV core, E1 and E2, p7, NS2, respectively was determined by quantitative measurement of core and E2 proteins by enzyme-linked immunoassay (ELISA). Packaging cells (4.5×10^6^) were lysed in 1.5 mL of PBS supplemented with 1% Triton X-100 and core protein abundance was determined using the ARCHITECT HCV Core AG Test (Abbott, Wiesbaden, Germany) according to the instructions of the manufacturer. For detection of HCV E2, packaging cells (4.5×10^6^) were lysed in 1.5 mL Ripa buffer (0.3 M NaCl, 20 mM Tris-HCl (pH 8), 1% sodium desoxycholate, 0.1% sodium dodecyl sulphate, 1% Triton X-100 and protease inhibitors (Roche, Mannheim). Total protein content of cell lysates was determined by a standard Bradford assay. Subsequently equal quantities of cell lysates were analyzed as described elsewhere [70]. In brief, Maxisorb mircrotiter plates (Nunc, Langenselbold, Germany) were coated with 500 ng/well *Galanthus nivalis* (GNA, Sigma-Aldrich, Steinheim, Germany) and blocked in blocking buffer consisting of 2.5% non-fat dry milk, 2.5% normal goat serum, 20 mM Tris-HCl (pH 7.5), 150 mM NaCl and 0.1% Tween-20. Cell lysates were bound on the GNA coated plates prior to incubation with the primary antibody (AP-33 [71]) at concentrations of 10 µg/well. After extensive washing with TBST (20 mM Tris-HCl pH 7.5, 150 mM NaCl and 0.1% Tween-20) the bound primary antibodies were detected using an AP-conjugated secondary antibody and 4-nitrophenyl phosphate disodium salt hexahydrate (Sigma-Aldrich, Steinheim, Germany). Absorbance was measured at 405 nm and 570 nm using a BioTek Synergy 2 plate reader (BioTek, Winooski, Vermont, USA) and the Gen5 1.08 software (BioTek).

To directly compare E2 expression between individual packaging cell lines, we performed a linear regression analysis of the relation between the Log OD value and the Log of the reciprocal dilution of the cell lysate. Based on this analysis we calculated the reciprocal dilution of the cell lysate required for an OD value of 0.5 in the ELISA assay, which reflects the relative expression of E2 between the given cell lysates.

### CD81-mediated neutralization of HCV infection

Infectivity of HCV particles produced from heterokaryons was inhibited with monoclonal antibodies against CD81. Huh-7.5 cells were pre-incubated with either α-CD81 specific antibodies (JS-81, Becton-Dickinson, Heidelberg, Germany) or α-CD13 antibodies (Becton-Dickinson, Heidelberg, Germany) as a negative control at a concentration of 5 µg/mL in DMEM for 30 min prior to infection as previously described [28]. Luciferase activity was determined 72 h post inoculation as described above.

### Virus replication und influence of human or mouse IFN-α

Huh-7.5 cells or Hep56.1D cells were seeded onto a 12-well plate. The next day cells were pre-incubated with either recombinant human (0–100 U/mL, hIFN-α-2b, IntronA, Essex Pharma, München, Germany) or mouse IFN-α (0–100 U/mL, mIFN-α-1, PBL Interferon Source, Piscataway, USA) for 4 h prior to infection with Luc-Jc1 or Luc-VSV, respectively. Four hours later medium was changed and IFN-α treatment resumed. Replication was determined 72 h post-infection by analyzing luciferase reporter activity in infected cells as described above.

### Heterokaryon formation induced by foamy virus glycoprotein expression and virus titration

Huh-7 Lunet N cells lacking CD81 [34] were co-cultured with the indicated naive cell lines or the transduced cell lines expressing human CD81 at a cell density of 1.5×10^5^ cells in a 12-well culture plate. The ratio of the different cell lines to Huh-7 Lunet N cells was adjusted according to the respective growth efficiency and ranged between 1∶1 and 1∶2. Fusion between these cells was initiated the next day by transfection of the co-cultures with a highly fusogenic variant of the prototype foamy virus envelope protein pczHFVenvEM066 [63] using lipofectamine 2000 (Invitrogen). Cells were challenged 30 hours later with infectious HCV particles produced in cell culture at a multiplicity of infection (MOI) of 2.3. To remove any unbound HCV particles left from inoculation, cells were washed with PBS 12 hours after inoculation. Cell entry, RNA replication and virus production was quantified by determining infectivity titers (TCID_50_/mL) in the culture fluids of these cells 48 h post inoculation as described above.

### Assessment of functional viral pattern recognition pathways in mouse liver cells

Given mouse liver cell lines were mock treated or transfected with 500 ng 5′triphosphorylated genomic RNA of VSV or with 10 µg poly(I:C) per well of a 6-well plate using Metafectene (Biontex, Martinsried, Germany) according to the instructions of the manufacturer. Alternatively, cells were challenged with recombinant La Crosse virus lacking the non-structural protein NSs (rLACVdelNSs;[32]) at a MOI equal to 5. Cells were incubated for 16 h at 37°C and harvested. Total cellular RNA was extracted using the RNeasy Mini kit (Qiagen, Hilden, Germany) according to the instruction of the manufacturer. Transcript levels of mouse ISG56 and IFN-β were analyzed using the QuantiTect SYBR Green PCR Kit in combination with QuantiTect Primer Assay (Qiagen) and the following primers: Qiagen Mm_Ifit1_1_SG  =  QT01161286 (mouse ISG56), Qiagen Mm_Ifnb1_1_SG  =  GT00249662 (mouse IFN-β). In total 100 ng of cDNA was used for real time PCR in a final reaction volume of 25 µL using an initial 15 minute denaturation at 95°C and 40 cycles consisting of 15 seconds denaturation at 94°C, 30 seconds annealing at 55°C and 30 seconds extension at 72°C. Signals of inducible mRNAs were normalized to the GAPDH mRNA (mouse GAPDH: Qiagen Mm_Gapdh_3_SG  =  GT01658692) using the ΔΔCt method [72]. Values of mock treated cells were set to 1. Secretion of IFN was monitored using a luciferase-based reporter gene assay. Briefly, supernatants were treated with benzonase for 2 h at 37°C to destroy inducer RNA. Alternatively supernatant was incubated with β-propiolactone (Acros Organics, Geel, Belgium) at 4°C over night followed by 2 h at 37°C for inactivation of the rLACdelNSs inducer virus. These inducer-cleared supernatants were incubated for 16 h with murine embryonic fibroblasts that had been transfected with 250 ng Mx1-Luc [73] and 25 ng pRL SV40 (Promega, Mannheim, Germany) reporter plasmids using Nanofectin (PAA, Cölbe, Germany). Cells were lysed and luciferase activity was determined using the Promega Dual Luciferase assay according to the instruction of the manufacturer. Recombinant, pan-specific IFN-α (IFN-α B/D BgIII, PBL Biomedical Laboratories, Piscataway, USA) served as standard.

## Supporting Information

Figure S1 Quantification of E2 expression in packaging cell lines. Lysates of given packaging cell lines were normalized for equal total protein content, serially diluted and incubated with *galanthus nivalis* lectin coated culture plates to capture glycosylated proteins. Bound viral E2 protein was detected using an E2-specific monoclonal antibody (AP33). In each case, lysates of the parental cell line served as negative control. The OD value was plotted against the reciprocal dilution of the cell lysate.
(TIF)

Figure S2 Comparable cell to cell fusion of different packaging cell lines. Indicated packaging cell lines were co-cultured with Huh-7.5 replicon cells. Fusion between co-cultured cells was induced by PEG-treatment. Fusion efficiency was quantified by counting the number of cells expressing both NS5A (replicon-derived) and E2 (lentiviral expression) per total number of cells. In total five independent microscopic views were evaluated including at least 1,000 cells. Fusion efficiency is given as % of cells displaying NS5A and E2 among the total cell population. Mean values including the standard deviation is given.
(TIF)

Figure S3 Efficient induction of IFN-β and ISG56 gene expression as well as IFN secretion in mouse liver cells by viral pathogen associated molecular patterns. Given mouse cells were either transfected with 5′triphosphorylated VSV RNA, poly(I:C) or infected with a recombinant La Crosse virus lacking the nonstructural protein NSs (LACVdelNSs;[32]). Sixteen hours later, cells and culture fluids were collected and (A) IFN-β and (B) ISG56 gene expression were assessed by quantitative reverse transcriptase polymerase chain reaction. (C) IFN secretion was determined using a luciferase-based reporter assay as described in the experimental procedures. Mean values of three independent experiments including the standard deviations are given.
(TIF)

Table S1 Overview of packaging cell lines used in this study.
(DOC)

Table S2 Overview of origin and receptor expression of cell lines used in this study. h: human; m: mouse, SR-BI: scavenger receptor class B type 1, CLDN1: claudin-1, OCLN: occludin.
(DOC)
