# Supplementary figures and images for: Completion of Hepatitis C Virus Replication Cycle in Heterokaryons Excludes Dominant Restrictions in Human Non-liver and Mouse Liver Cell Lines
Source: PLoS Pathog. 2011 Apr 28;7(4):e1002029. doi: 10.1371/journal.ppat.1002029 (PMC3084199; doi:10.1371/journal.ppat.1002029)

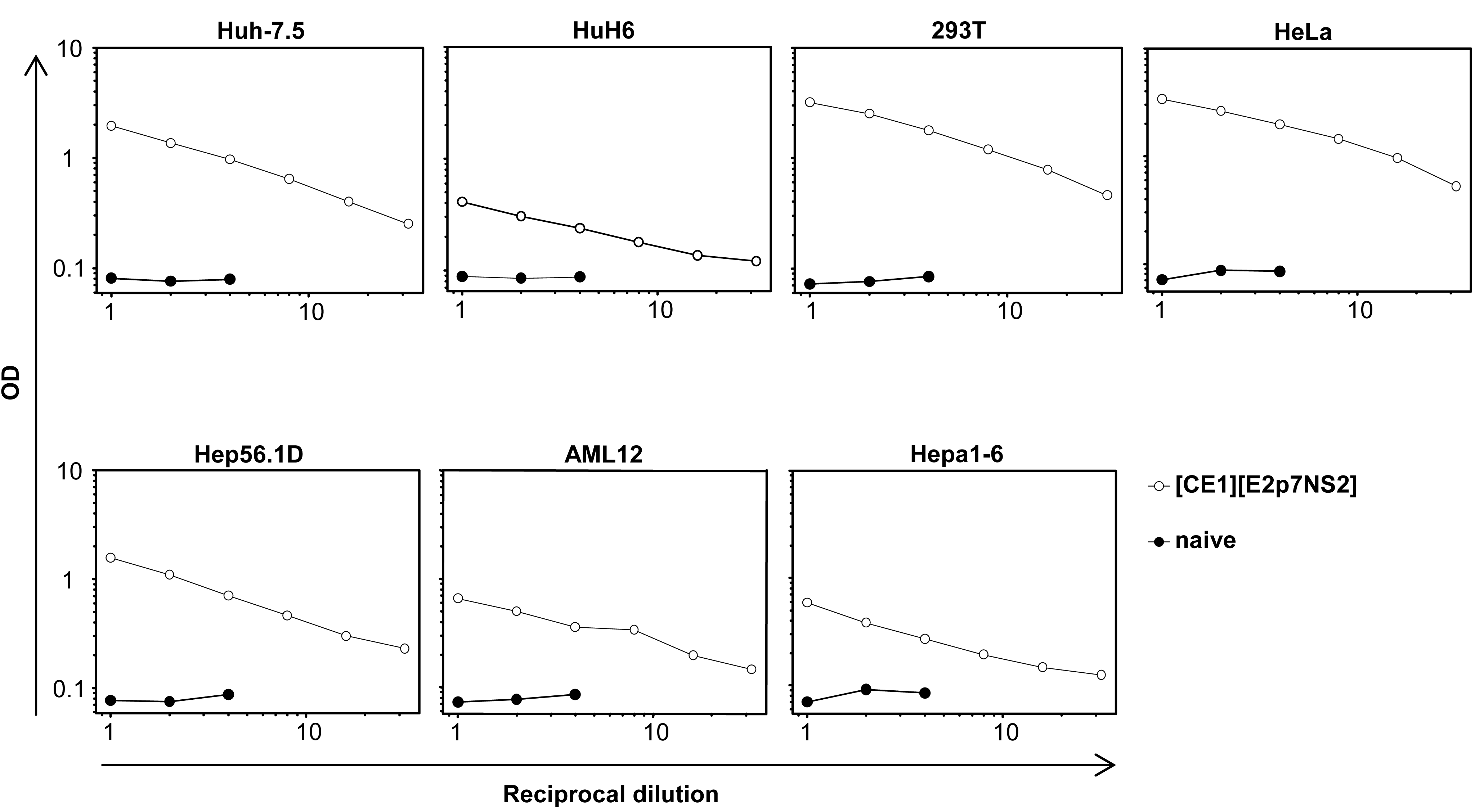

Supplement: Figure S1 — Quantification of E2 expression in packaging cell lines. Lysates of given packaging cell lines were normalized for equal total protein content, serially diluted and incubated with galanthus nivalis lectin coated culture plates to capture glycosylated proteins. Bound viral E2 protein was detected using an E2-specific monoclonal antibody (AP33). In each case, lysates of the parental cell line served as negative control. The OD value was plotted against the reciprocal dilution of the cell lysate. (TIF) [file ppat.1002029.s001.tif]

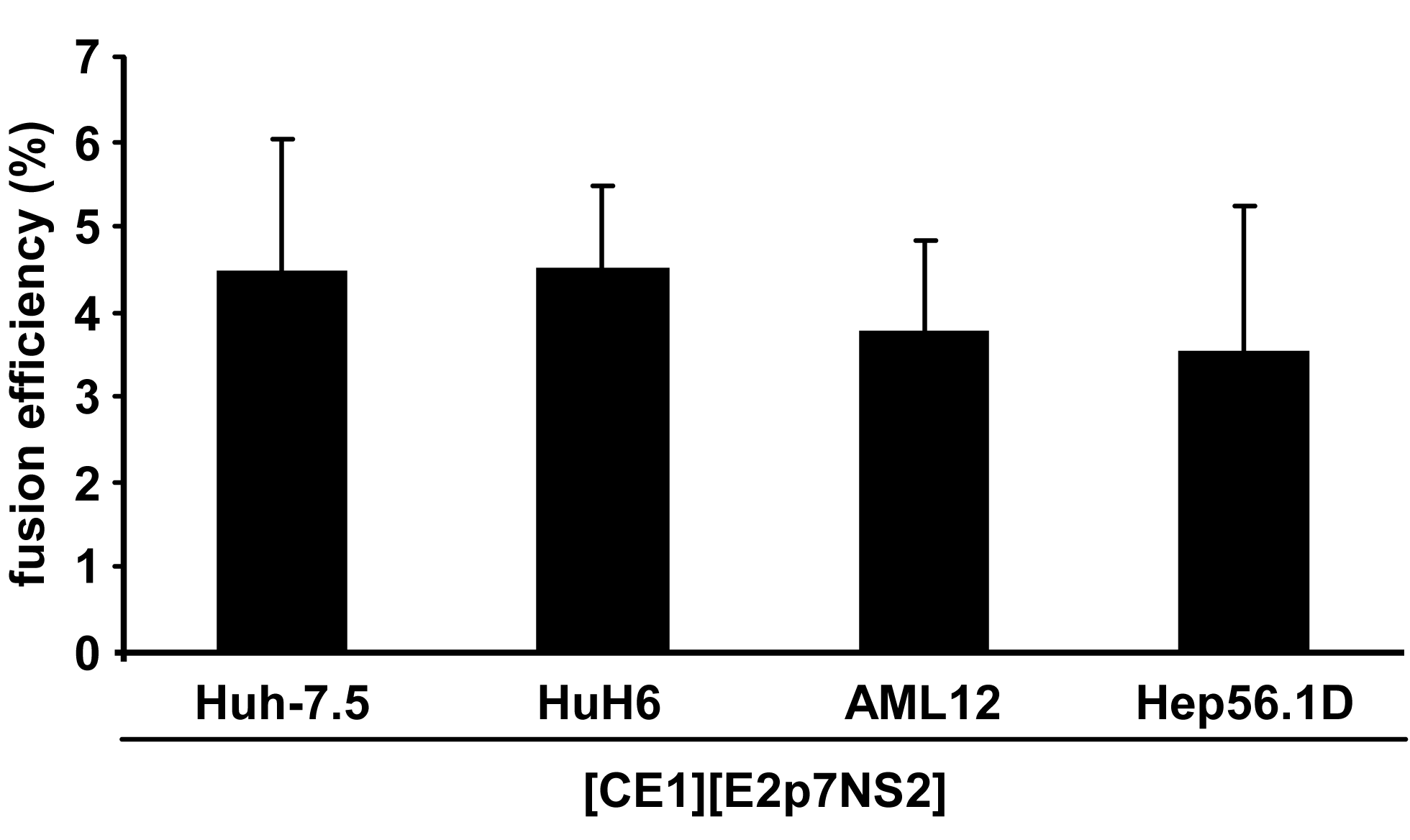

Supplement: Figure S2 — Comparable cell to cell fusion of different packaging cell lines. Indicated packaging cell lines were co-cultured with Huh-7.5 replicon cells. Fusion between co-cultured cells was induced by PEG-treatment. Fusion efficiency was quantified by counting the number of cells expressing both NS5A (replicon-derived) and E2 (lentiviral expression) per total number of cells. In total five independent microscopic views were evaluated including at least 1,000 cells. Fusion efficiency is given as % of cells displaying NS5A and E2 among the total cell population. Mean values including the standard deviation is given. (TIF) [file ppat.1002029.s002.tif]

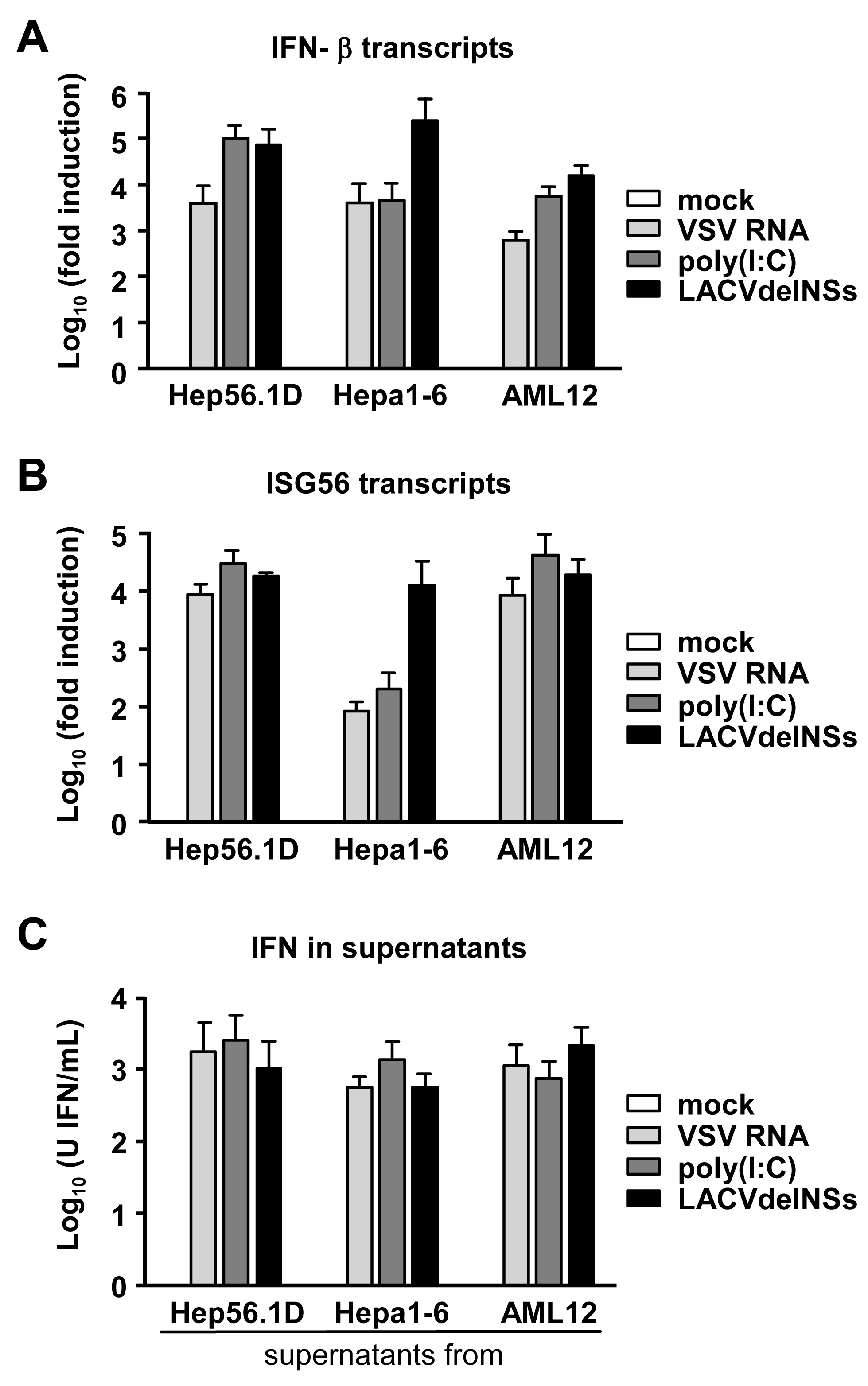

Supplement: Figure S3 — Efficient induction of IFN-β and ISG56 gene expression as well as IFN secretion in mouse liver cells by viral pathogen associated molecular patterns. Given mouse cells were either transfected with 5′triphosphorylated VSV RNA, poly(I:C) or infected with a recombinant La Crosse virus lacking the nonstructural protein NSs (LACVdelNSs;[32]). Sixteen hours later, cells and culture fluids were collected and (A) IFN-β and (B) ISG56 gene expression were assessed by quantitative reverse transcriptase polymerase chain reaction. (C) IFN secretion was determined using a luciferase-based reporter assay as described in the experimental procedures. Mean values of three independent experiments including the standard deviations are given. (TIF) [file ppat.1002029.s003.tif]
