# Supplementary material for: Completion of Hepatitis C Virus Replication Cycle in Heterokaryons Excludes Dominant Restrictions in Human Non-liver and Mouse Liver Cell Lines
Source: PLoS Pathog. 2011 Apr 28;7(4):e1002029. doi: 10.1371/journal.ppat.1002029 (PMC3084199; doi:10.1371/journal.ppat.1002029)
Supplement: Table S1 — Overview of packaging cell lines used in this study. (DOC) [file ppat.1002029.s004.doc]

| **Species** | **Cell line** | **Selection** | **Description** |
| --- | --- | --- | --- |
| **Human** | Huh-7.5 [CE1][E2p7NS2] | Blasticidin  5 µg/mL | Cell lines derived from parental cell lines, stably expressing viral proteins core, E1, E2, p7, and NS2, transduced by lentiviral gene transfer of two independent gene cassettes with one selectable marker |
|  | HuH6 [CE1][E2p7NS2] | Blasticidin  5 µg/mL |  |
|  | 293T  [CE1][E2p7NS2] | Blasticidin  2.5 µg/mL |  |
|  | HeLa  [CE1][E2p7NS2] | G418  750 µg/mL;  Blasticidin  5 µg/mL | Cell lines derived from parental cell lines, stably expressing viral proteins core, E1, E2, p7, and NS2, transduced by lentiviral gene transfer of two independent gene cassettes with different selectable markers |
| **Mouse** | Hep56.1D  [CE1][E2p7NS2] | G418  750 µg/mL;  Blasticidin  5 µg/mL |  |
|  | AML12  [CE1][E2p7NS2] | G418  750 µg/mL;  Blasticidin  5 µg/mL |  |
|  | Hepa1-6  [CE1][E2p7NS2] | G418  750 µg/mL;  Blasticidin  5 µg/mL |  |
