# Supplementary material for: Completion of Hepatitis C Virus Replication Cycle in Heterokaryons Excludes Dominant Restrictions in Human Non-liver and Mouse Liver Cell Lines
Source: PLoS Pathog. 2011 Apr 28;7(4):e1002029. doi: 10.1371/journal.ppat.1002029 (PMC3084199; doi:10.1371/journal.ppat.1002029)
Supplement: Table S2 — Overview of origin and receptor expression of cell lines used in this study. h: human; m: mouse, SR-BI: scavenger receptor class B type 1, CLDN1: claudin-1, OCLN: occludin. (DOC) [file ppat.1002029.s005.doc]

| **Species** | **Parental**  **cell line** | **Origin** | **Receptor expression** |
| --- | --- | --- | --- |
| Human | Huh-7 Lunet N | Subclone of Huh-7 hepatoma cell line [1] | SR-BI  CLDN1  OCLN |
|  | Huh-7.5 | Subclone of Huh-7 hepatoma cell line [2] | CD81  SR-BI  CLDN1  OCLN |
|  | HuH6 | Hepatoblastoma [3,4] | CD81  SR-BI  OCLN |
|  | 293T | Human embryonic kidney transformed with adenovirus 5 DNA (ATCC# CRL-11268) | CD81  SR-BI  OCLN |
|  | HeLa | Cervical adenocarcinoma cell line (ATCC# CCL-2) | CD81,  SR-BI |
| Mouse | Hep56.1D | Primary hepatocellular carcinoma (kind gift of J. Encke)  Adult C57BL/6J mice | mCD81, mSR-BI,  mCLDN1 (low level expression),  mOCLN not detectable by Western blot analysis |
|  | AML12 | Non-transformed hepatocyte cell line transgenic for TGF-α (CD1 strain, line MT42) |  |
|  | Hepa1-6 | derivative of the BW7756 mouse hepatoma (ATCC# CRL-1830)  C57/L mice |  |
| **Species** | **Cell line** | **Origin** | **Receptor expression** |
| Mouse | Hep56.1D hCD81 | Cell lines derived from parental cell lines, stably expressing human CD81 transduced by lentiviral gene transfer | Mouse receptors + hCD81  (selected by Blasticidin 5 µg/mL) |
|  | AML12  hCD81 |  |  |
|  | Hepa1-6 hCD81 |  |  |

**SUPPLEMENTAL REFERENCES**

1. Witteveldt J, Evans MJ, Bitzegeio J, Koutsoudakis G, Owsianka AM, et al. (2009) CD81 is dispensable for hepatitis C virus cell-to-cell transmission in hepatoma cells. J Gen Virol 90: 48-58.

2. Blight KJ, McKeating JA, Rice CM (2002) Highly permissive cell lines for subgenomic and genomic hepatitis C virus RNA replication. J Virol 76: 13001-13014.

3. Doi I (1976) Establishment of a cell line and its clonal sublines from a patient with hepatoblastoma. Gann 67: 1-10.

4. Windisch MP, Frese M, Kaul A, Trippler M, Lohmann V, et al. (2005) Dissecting the interferon-induced inhibition of hepatitis C virus replication by using a novel host cell line. J Virol 79: 13778-13793.
